# Incidence of and Factors Associated with False Positives in Laboratory Diagnosis of Norovirus Infection by Amplification of the RNA-Dependent RNA Polymerase Gene

**DOI:** 10.1371/journal.pone.0109876

**Published:** 2014-09-29

**Authors:** Fang-Ru Lin, Yu-Hua Shen, Chun-Wan Fang, Shian-Sen Shie, Chung-Guei Huang, Shuan Yang, Shu-Li Yang, Kuo-Chien Tsao, Yhu-Chering Huang, Ming-Wei Lai, Chih-Jung Chen

**Affiliations:** 1 College of Medicine, Chang Gung University, Taoyuan, Taiwan; 2 Department of Laboratory Medicine, Chang Gung Memorial Hospital, Taoyuan, Taiwan; 3 Division of Infectious Diseases, Department of Internal Medicine, Chang Gung Memorial Hospital, Taoyuan, Taiwan; 4 Division of Pediatric Infectious Diseases, Department of Pediatrics, Chang Gung Memorial and Children’s Hospital, Taoyuan, Taiwan; 5 Division of Pediatric Gastroenterology, Department of Pediatrics, Chang Gung Memorial and Children’s Hospital, Taoyuan, Taiwan; University of Houston, United States of America

## Abstract

**Background:**

Conventional reverse transcription-polymerase chain reaction (RT-PCR) amplification of the RNA-dependent RNA polymerase (RdRp) gene remains a used method for the rapid detection of norovirus (NV) in clinical laboratories. The incidence of and factors associated with false positives in this assay have not been previously evaluated.

**Methods/Principal Findings:**

After an NV outbreak caused by the GII.4 Sydney strain in 2012, we reanalysed 250 stool samples positive for NV by RdRp gene detection. True positives were confirmed in 154 (61.6%) samples by successful amplification and sequencing confirmation of the viral protein 1 gene. Of the remaining 96 samples that underwent RT-PCR for the RdRp gene, 34 samples yielded PCR products of the expected length. However, the sequences of the amplicons belonged to the human genome, with 91–97% matched nucleotide sequences, indicating false positives. Multivariate analysis of the clinical features of the patients identified a positive stool culture for bacteria (adjusted odds ratio [aOR] 9.07, 95% adjusted confidence interval [aCI] 2.17–37.92, *P* = .003) and the use of parenteral antibiotics (aOR 5.55, 95% aCI 1.21–24.73, *P* = .027) as significant and independent factors associated with false positives.

**Conclusion:**

Conventional RT-PCR targeting the RdRp gene of NV can lead to false positives in patients with bacterial enterocolitis by incidental amplification of DNA from a human source.

## Introduction

Norovirus (NV) is now recognised as the leading cause of acute non-bacterial gastroenteritis and is frequently responsible for food-borne gastroenteritis outbreaks worldwide [1], [2]. NV-associated disease in previous healthy individuals is self-limiting and usually characterised by vomiting, watery diarrhoea, abdominal cramps and low-grade fever. Severe outcomes, such as hospitalisation and mortality, are uncommon and estimated to occur in 0.54% and 0.06%, respectively, of infected cases during NV outbreaks [3]. Young children, immunocompromised hosts and institutionalised elderly are the populations most vulnerable to severe NV infections [4].

NV possesses a 7.5 kb positive single-stranded polyadenylated RNA genome with 3 open-reading frames (ORF1-3). Phylogenetic analysis of the genome classified NV into five major genogroups, of which genogroup I (GI), GII and GIV are associated with human diseases [5]. Due to the lack of a cell culture system for virus propagation and the unavailability of a commercialised kit for rapid diagnosis, the timely identification of NV in clinical laboratories is mainly dependent on the detection of viral nucleic acids using reverse transcription-polymerase chain reaction (RT-PCR) or a hybridisation assay [5]–[7]. A variety of RT-PCR methods has been developed and proven to have good sensitivity in detecting distinct genogroups of epidemic NV strains [6], [8]–[10]. The genome regions encoding RNA-dependent RNA polymerase (RdRp) in ORF1 and viral protein 1 (VP1), a capsid protein, in ORF2 are the most common RT-PCR targets in the rapid diagnosis of NV infections. For instance, the region in ORF2 has been used as the target site for RT-PCR detection of norovirus by CaliciNet, a national laboratory surveillance network for norovirus outbreaks coordinated by CDC [1]. RdRp is a highly conserved region in the NV genome, and primer sets designed to amplify this allele have been shown to be able to detect NV with satisfactory sensitivity [11]. The assay remains used in the diagnosis of NV infection in clinical laboratories, surveillance studies and epidemiology studies [7], [12], [13].

In 2012, we encountered a major outbreak of acute gastroenteritis associated with NV of a subgenogroup (GII.4, Sydney strain) in Taiwan [14]. The conventional RT-PCR method targeting the RdRp gene was applied for the rapid detection of NV in faecal samples during this outbreak [11]. We made the unusual observation that a substantial proportion of NV-positive patients in this outbreak had manifestations mimicking bacterial colitis, which included high fever, bloody stool, leucocytosis in the peripheral blood and highly elevated levels of inflammatory markers in the serum. A preliminary investigation of the clinical features of patients with unusual presentations and the detection of NV suggested that laboratory error might have been accountable for the atypical presentation of the NV infections. A number of patients with acute gastroenteritis in this outbreak might have been falsely reported as having NV infection. To better understand this event, we conducted this study to reanalyse the residual stool samples and review the medical information of the affected patients in the 2012 outbreak. The results of this study allowed a comprehensive evaluation of the incidence of and factors associated with false positivity in the RT-PCR assay in the laboratory diagnosis of human NV infections.

## Methods

### Ethics statement

The study was approved by the institute review boards from Chang Gung Memorial Hospital in August 2013, which allowed the genotypic characterizations of the norovirus isolates and review of the medical data of the patients. A waiver of consent was granted given the retrospective nature of the project and anonymous analysis of the clinical information of patients.

### Study design

From December 1, 2011, to December 31, 2012, a list of patients whose stools had been sent for NV detection was retrieved from an electronic database maintained by the clinical virology laboratory of Chang Gung Memorial Hospital. The electronic database contained records of patients’ names; chart numbers; dates of sample collection; NV detection results; and, if positive, the genogroup of NV by RT-PCR targeting of the RdRp gene (discussed below). The hospital was located in northern Taiwan and provided primary to tertiary care to patients, including children of all ages. Residual faecal samples were routinely stored at 0°C after extraction of nucleic acids. In the present study, 250 (22.4%) of 1115 NV-positive patients were randomly selected from the patient list, and their residual faecal samples were obtained from the clinical laboratory for further testing for confirmation of NV. The medical information of the patients was collected by chart review and included demographics, clinical presentations, laboratory values, treatments and outcomes (Table 1). Data from microscopy studies and bacterial cultures of stool, if available, were also collected. The study was approved by the institutional review board of Chang Gung Memorial Hospital.

**Table 1:** Univariate analysis of the demographics and clinical parameters associated with false-positive results in the laboratory diagnosis of norovirus infections using RT-PCR targeting the RdRp gene.

Factor	All episodes (n = 152)	True positives (n = 124)	False positives (n = 28)	*P*
**Demographics**				
Male gender (%)	59.2	61.3	50.0	.272
Age (years)	3.3±3.3	3.1±2.9	4.0±4.4	.296
Body weight (kg)	16.5±17.5	16.7±18.7	15.9±10.8	.843
Breast feeding (%)	30.9	33.1	21.4	.324
Rotavirus vaccination (%)	14.5	15.3	10.7	.767
Household member having diarrhoea (%)	23.0	25.0	14.3	.224
**Underlying diseases**				
Any atopic disease	27	29	17.9	.229
Renal insufficiency (%)	.7	.8	0	1.000
Developmental delay (%)	4.6	5.6	0	.350
Congenital heart diseases (%)	2.6	1.6	7.1	.155
Immunodeficiency (%)	.7	.8	0	1.000
Malignant solid tumour (%)	.7	0	3.6	.184
**Clinical features**				
Hospital stay (d)	5.0±5.4	4.8±5.8	5.8±3.5	.386
Fever (%)	67.8	61.2	92.9	.002
Fever duration (d)	2.0±2.2	1.5±1.8	3.9±2.9	<.001
Vomiting (%)	81.6	87.9	53.6	<.001
Diarrhoea (%)	93.4	92.7	96.4	.690
Max no. of stools (/d)	5.5±3.5	4.8±3.2	8.3±3.2	<.001
Diarrhoea duration (d)	5.5±3.6	5.1±3.5	7.2±3.7	.006
Bloody stool (%)	16.4	8.1	53.6	<.001
Convulsion (%)	5.9	6.5	3.6	1.000
Use of parenteral antibiotics (%)	26.3	16.1	72.4	<.001
**Laboratory data**				
Stool study				
Occult blood (%)	25.7	19.2	59.3	<.001
Pus cells (%)	8.6	3.3	33.3	<.001
Mucus (%)	19.3	13.3	51.9	<.001
Positive rotavirus Ag (%)	3.9	4.2	3.6	1.000
Haemogram and biochemical study				
White blood cell count (/µl)	11,803±6,589	11,975±6,773	11,050±5,773	.505
Neutrophil count (/µl)	7,393±5,702	7,747±5,847	5,861±4,659	.115
Immature neutrophil count (/l)	273±622	219±573	508±769	.070
Haemoglobin (g/µl)	12.5±1.5	12.7±1.5	12.1±1.0	.053
C-reactive protein (mg/l)	25.0±47.7	17.6±37.5	70.8±72.5	.001
Positive stool bacterial culture (%)	14.5	6.9	53.6	<.001
Positive blood bacterial culture (%)	2.0	2.1	3.7	.525

### Preparation of stool samples and viral RNA extraction

During the period of study, a LabTurbo Mini Kit (Cat. No. LVN480-300, Taigen Bioscience Corporation, Taipei, Taiwan) was used to extract viral RNA in the clinical virology laboratory. The procedure was accomplished with the automatic nucleic acid extraction system LabTurbo 48 Compact (Taigen Bioscience Corporation, Taipei, Taiwan). In the current study, we repeated viral RNA extraction from the 250 residual faecal samples using the QIAamp Viral RNA Mini Kit (Qiagen, Hilden, Germany) according to the manufacturer’s instructions. The extracted RNA samples were stored at 0°C before undergoing RT-PCR.

### Reverse transcription

cDNA synthesis was performed using the SuperScript III First-Strand Synthesis System (Invitrogen, Life Technologies, NY, USA) according to the instructions of the manufacturer. Briefly, 8 µl of the RNA was mixed with 1 µl of random hexamer (50 ng/µl) and 1 µl of 10 mM dNTP mix at 65°C for 10 min and cooled on ice for 1 min. The cDNA synthesis mix was incubated with 1 µl of Superscript III RT buffer (200 U/µl), 2 µl of 0.1 M DTT, 1 µl of 40 U RNA-OUT, 4 µl of 25 mM MgCl_2_ and 2 µl of 10x RT buffer. RT was performed at 25°C for 10 min, followed by 50°C for 50 min and 85°C for 5 min, and then cooled down on ice for 10 min. In the final step, 1 µl of RNase H was added to the mixture and incubated at 37°C for 20 min.

### PCR amplification of RdRp gene

The detection of NV in the clinical virology laboratory during the period of study was routinely performed by amplification of part of the RdRp gene using a procedure described elsewhere [11]. Two primer sets, G-1 and G-2, were used to detect genogroups I (GI) and II (GII), respectively, of NV. Both the G-1 and the G-2 primer sets shared the same primer, SR33 (TGTCACGATCTCATCATCACC), for negative-strand cDNA synthesis. For positive-strand synthesis, the G-1 set contained three primers, SR48 (GTGAACAGCATAAATCACTGG), SR50 (GTGAACAGTATAAACCACTGG) and SR52 (GTGAACAGTATAAACCATTGG), whereas the G-2 set contained one primer, SR46 (TGGAATTCCATCGCCCACTGG). The purpose of using multiple primers in G-1 set was to overcome the obstacle of poor primer annealing due to diverse nucleotide sequence in the target site. SR48, SR50, SR52 and SR46 are located at the same position in the RdRp gene and are expected to generate a PCR product of 123 bp for NV of both genogroups. The PCR conditions were as follows: one cycle of denaturation at 94°C for 3 min; 40 amplification cycles with denaturation for 1 min at 94°C, annealing for 1 min 30 sec at 50°C and extension for 2 min at 60°C; and a final cycle of incubation at 72°C for 7 min.

### PCR amplification of VP1 gene

Using procedures described elsewhere [6], for NV GI PCR, the primers G1SKF (CTGCCCGAATTYGTAAATGA) and G1SKR (CCAACCCARCCATTRTACA) were used, and for NV GII PCR, the primers G2SKF (CNTGGGAGGGCGATCGCAA) and G2SKR (CCRCCNGCATRHCCRTTRTACAT) were used. The PCR conditions were as follows: one cycle of denaturation at 95°C for 15 min; 40 amplification cycles with denaturation for 1 min at 94°C, annealing for 1 min at 58°C and extension for 30 sec at 72°C; and a final cycle of incubation at 72°C for 10 min.

### Sequencing of RdRp and VP1 amplicon and phylogenetic analysis of VP1 sequences

The primer SR33 was used for sequencing of the RdRp amplicon. The primers G1SKF and G2SKF were used for sequencing of the VP1 amplicons of GI and GII NV, respectively. The sequencings were performed with an ABI 3730 DNA Analyzer (Applied Biosystems, Life Technologies, NY, USA). The sequences of the VP1 amplicons were additionally inputted into and cleaned in DNASTAR (DNASTAR, Inc., Madison, WI, USA), and phylogenetic trees were constructed using the Tamura-Nei model in MEGA 4 (The Biodesign Institute, Tempe, AZ, USA).

### Statistics

A comparison of categorical variables between false-positive and true-positive cases was performed using a chi-square test or Fisher’s exact test, where appropriate, whereas differences between the two groups in numerical variables were tested by a two-sample t test. Multiple logistic regression analysis was applied to explore factors associated with false positivity. Statistical significance was defined as a *P* value of <0.05 in the tests. The data were analysed with SPSS Statistics for Windows, version 20.0 (IBM Corp., Armonk, NY, USA).

## Results

**Figure 1:**
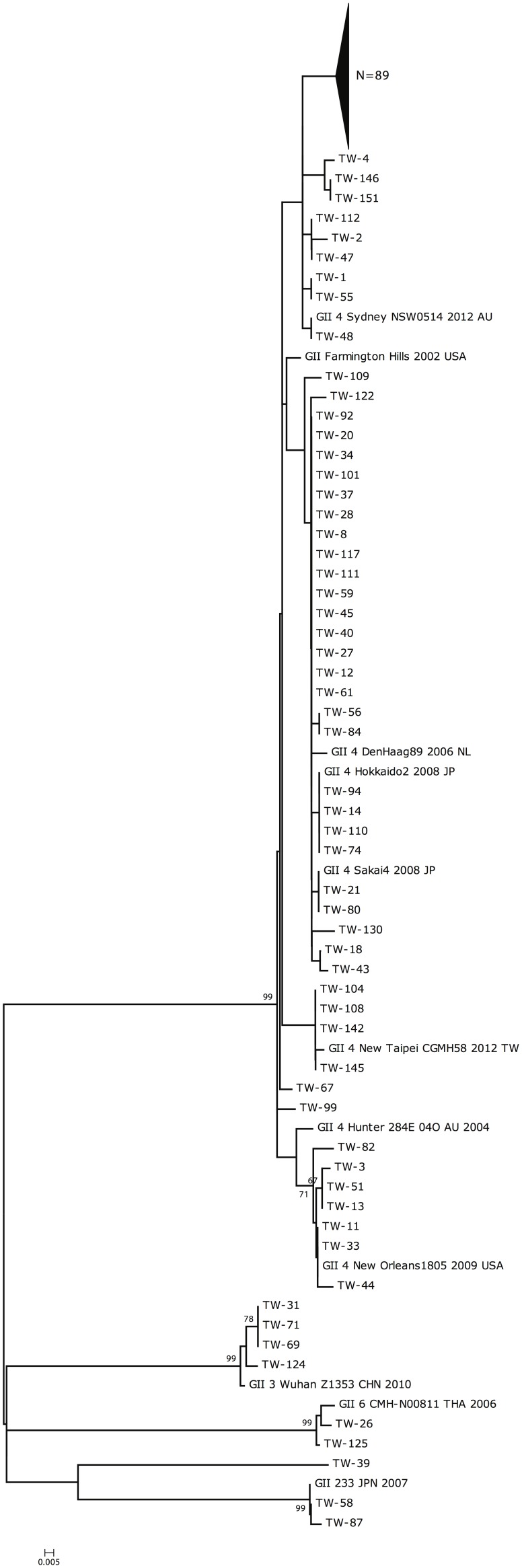
Phylogenetic analysis of viral protein 1 sequences from 154 strains of norovirus. Footnote of Figure 1: A total of 11 reference norovirus strains were included in the analysis. Except for strains of GII3 Wuhan Z1353 CHN2010, GII 6 CMH-N00811 THA 2006 and GII 233 JPN 2007, the other 8 reference strains were close related to the GII 4 Sydney strains.

**Figure 2:**
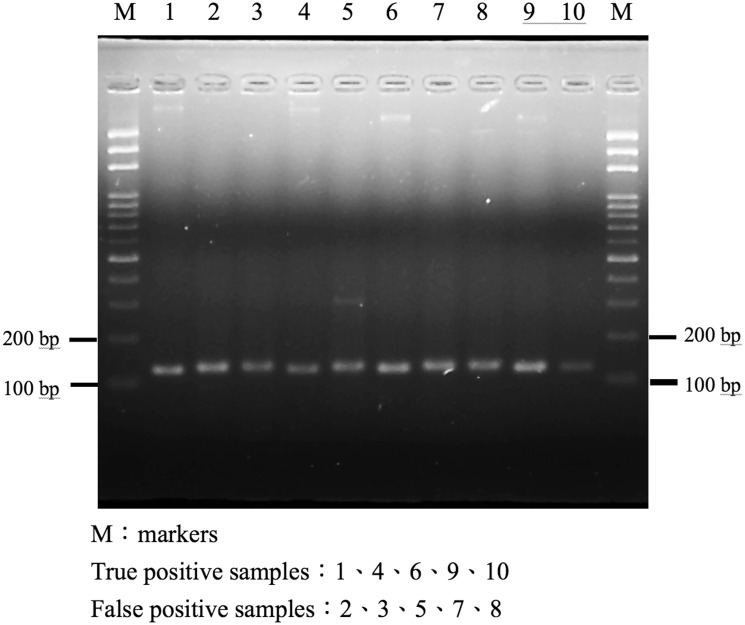
Gel electrophoresis of PCR products by RT-PCR amplification of RdRp gene in true-positive samples and false-positive samples. Footnote of Figure 2: The 5 true-positive samples were confirmed by viral protein 1 gene amplificaion and sequence of amplicons. In the 5 false-positive samples, the amplicon sequences were belonged to human DNA.

Of the 250 faecal samples, PCR amplification of the VP1 gene was successful in 154 (61.6%) samples. Sequencing of the VP1 amplicon and phylogenetic analysis of the nucleotide sequences disclosed that a majority (145, 94.1%) of the 154 strains were of the GII.4 Sydney strain (Figure 1). Amplification of the RdRp gene was performed for the other 96 samples, 63 of which failed the PCR amplification. The remaining 33 (13.2%) samples yielded PCR products of the expected length (Figure 2). However, sequencing of the 33 RdRp amplicons disclosed that the sequences did not belong to the NV genome, but rather were closer to the human genome, with 91–97% matched nucleotide sequences (Table S1). The data clearly demonstrated that the RT-PCR method targeting the RdRp gene for NV diagnosis could incidentally amplify a human genome segment and resulted in false positivity in at least 13.2% of the 250 clinical stool samples.

**Figure 3:**
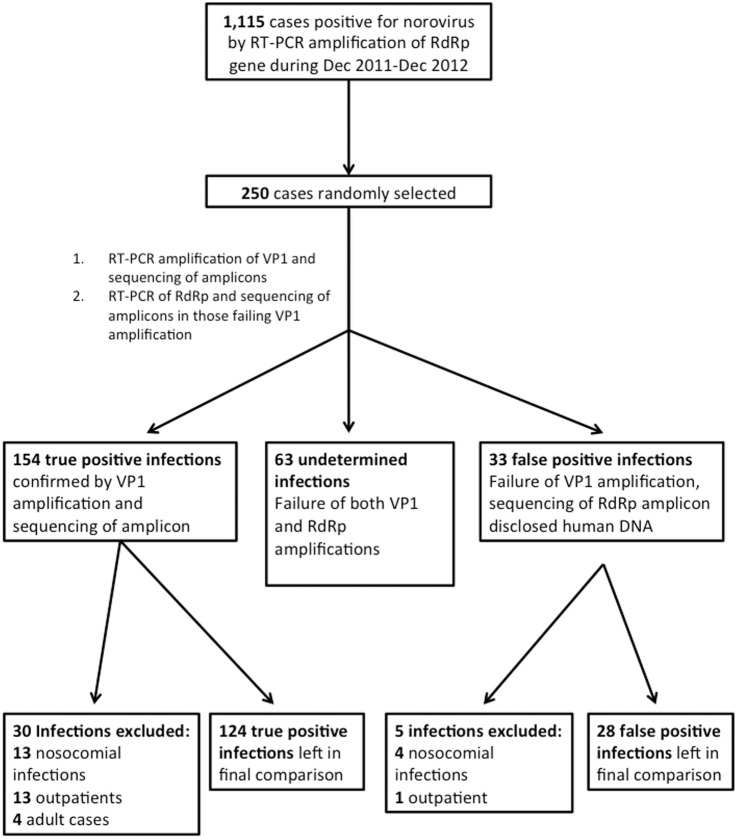
Flow chart of patients undergoing norovirus detections and selection of infection episodes for the identification of factors associated with false-positivity.

To investigate the factors associated with the false positivity of the assay, the clinical information of the patients was collected and compared between 154 true-positive infection episodes confirmed by VP1 sequencing and 33 false-positive episodes. To minimise heterogeneity between the two patient groups, we excluded adult patients (4 episodes), outpatients (14 episodes) and nosocomial infections after 48 hours of admission (17 episodes). A total of 152 episodes, including 124 true infections and 28 false-positive infections, were left in the final comparison (Figure 3).

The 152 episodes occurred in 152 paediatric patients, and 59.2% of them were male. The mean age was 3.3±3.3 years old. Common manifestations included fever (67.8%), vomiting (81.6%) and diarrhoea (93.4%). Convulsions occurred in 9 (5.9%) episodes. The variables that were correlated with false positivity in a univariate analysis are displayed in Table 1. There was no significant difference between the true infections and false-positive infections regarding patient demographics and underlying conditions. When compared with the patients with true infections, the patients with false-positive infections had a greater severity of gastroenteritis, as indicated by a greater incidence and longer duration of fever (*P* = .002 and *P*<.001, respectively), greater numbers of daily diarrhoea episodes (*P*<.001), higher chances of bloody stools (*P*<.001) and stool with mucus (*P*<.001) and a greater incidence of being treated with parenteral antimicrobial agents (*P*<.001) (Table 1). The greater severity of the false-positive infections was also supported by the laboratory data, which disclosed a higher immature neutrophil count in the peripheral blood (*P* = .070); higher levels of C-reactive protein (*P* = .001); and higher chances of occult blood (*P*<.001), pus cells (*P*<.001) and mucus (*P*<.001) in the microscopy examination of stool samples and a greater incidence of a positive stool culture for bacteria (*P*<.001). The bacterial organisms identified in stool included *Salmonella enteritidis* (9), *Campylobacter jejuni* (4), *Campylobacter coli* (1) and *Pseudomonas aeruginosa* (1) in false-positive infections and *Campylobacter jejuni* (3), *Salmonella enteritidis* (4) in true-positive infections.

Multivariate analysis (Table 2) identified a positive stool culture for bacteria (adjusted odds ratio [aOR] 9.07, 95% adjusted confidence interval [aCI] 2.17–37.91, *P* = .003) and the use of parenteral antibiotics (aOR 5.55, 95% aCI 1.21–24.72, *P* = .027) as significant and independent factors associated with false positivity in RdRp detection.

**Table 2:** Factors associated with false-positive results in the laboratory diagnosis of norovirus infection using RT-PCR targeting the RdRp gene, as determined by multiple logistic regression analysis.

Factor	Adjusted odds ratio	Adjusted 95% confidence interval	*P*
Vomiting	0.39	0.09–1.72	.215
Positive stool bacterial culture	9.07	2.17–37.92	.003
Pus in stool	6.33	0.85–47.29	.072
Occult blood	0.91	0.17–4.80	.910
Mucus in stool	1.12	0.19–6.73	.905
Total fever duration	1.39	0.99–1.97	.059
C-reactive protein	1.00	0.99–1.02	.554
Use of parenteral antibiotics	5.55	1.21–24.73	.027

## Discussion

The results of the current study clearly demonstrated that false-positive results could occur in the laboratory diagnosis of NV infection using the RT-PCR method targeting the RdRp gene. The false positivity was caused by nonspecific amplification of human genome segments and the generation of PCR products with a length similar to that of the expected RdRp amplicon. A stool culture positive for bacteria and the use of parenteral antibiotics were the most important factors associated with the false positivity of the assay. The data indicated that without further sequencing of the amplicon, NV could be possibly identified as the responsible or co-infecting pathogen in bacterial colitis by the RT-PCR assay targeting the RdRp gene.

The pathogenesis of bacterial colitis should largely explain its significant association with this laboratory error. Adherence to and subsequent invasion of the intestinal wall are the main mechanisms of enterocolitis caused by bacteria, including *Salmonella* and *Campylobacter* spp. [15]–[18]. Either direct invasion of the intestinal wall or the production of enterotoxin by the bacteria can lead to massive destruction of villous cells, with or without the extrusion of leucocytes into the intestinal lumen [16], [18], [19]. The incidental amplification of the human genome in this assay indicated that human DNA could not be completely eliminated from the stool samples during the process of viral RNA extraction. The existence of abundant nucleated human cells in the stool samples due to bacterial colitis further increased the chance of human genome contamination.

Use of parenteral antibiotic was another significant factor associated with false-positive result of the NV detection. Antimicrobial therapy was not recommended for routine treatment of bacterial colitis in children [20], [21]. However, antibiotic treatment is essential in patients with extra-intestinal infections, in immunocompromised hosts and may be of benefit in severely ill patients [22]. Previous experience in our institute further showed that the *Salmonella*-infected children with longer febrile duration and higher C-reactive protein levels were more frequently put on empirical antimicrobial therapy and had more complications compared to those without antibiotic treatment [23]. The observations suggested that the use of antimicrobial agents in this cohort was a surrogate of severe bacterial colitis which lead to false positive results with the above-mentioned mechanism.

Distinct from the pathogenesis of bacterial colitis, massive amounts of leucocytes and intestinal epithelial cells in stools are infrequent events in viral gastroenteritis. In this condition, the mucosa of the small intestine is usually intact, with the major histologic changes consisting of the blunting of villi and the shortening of microvilli, resulting in a transduction of fluid into the intestinal lumen [24]. These characteristics suggest that the false positivity of the assay might less frequently occur in patients with gastroenteritis caused by other viral aetiology, such as rotavirus or adenovirus. This speculation was also supported by the finding in this study that the incidences of rotavirus co-infection were similar between the patients with true positives and the patients with false-positive NV infections (Table 1).

The RT-PCR method targeting the RdRp gene that was used in the current study was developed by Ando et al in 1995. This method can detect NV with high sensitivity and readily differentiate the two major genogroups of NV [5], [11]. The assay had been widely implemented in laboratory diagnosis and various epidemiology studies of human NV infections [7], [12], [13], [25]. Misinterpretation of electrophoresis results due to the similar size of the PCR products between nonspecific amplification and virus-specific amplicons has not been reported for this assay, although the phenomenon has been recognised in other RT-PCR methods of NV detection [26]. Using a large number of amplification cycles (40 cycles in this study) during the PCR procedure may also increase the chance of nonspecific amplification and contribute to the false positivity. The use of a lower number of amplification cycles is expected to diminish the rate of false positives. However, this adjustment can also potentially compromise the sensitivity of this assay and limit its use in detecting this infecting agent, which has high genomic diversity.

A confirmatory test by sequencing of the amplicon or southern blot hybridisation can increase the specificity of the assay to nearly 100% and is the ultimate solution to this laboratory error [5], [26]. However, it is not practical to routinely sequence the PCR amplicon or to perform a southern blot for confirmation, especially in busy clinical laboratories. We believe that the laboratory error can also occur in other clinical settings and/or epidemiological studies of human subjects without performing confirmatory tests. Nevertheless, the method may still be useful in detecting NV in non-human samples, such as food, water or animal-derived samples, given its convenience and high sensitivity.

In conclusion, we identified a high false-positive rate (at least 13.2%) in the laboratory diagnosis of NV infection in stool samples using the conventional RT-PCR method targeting the RdRp gene. The RT-PCR can spuriously amplify segments of the human genome and generate amplicons of the expected molecular length that cannot be readily differentiated in an electrophoresis gel. Without a confirmatory test, this method should no longer be used for the rapid detection of NV in clinical samples.

## Supporting Information

Table S1 **Sequences and BLAST results of RdRp amplicons in 33 samples false-positive for norovirus.**
(PDF)
