# Supplementary material for: Incidence of and Factors Associated with False Positives in Laboratory Diagnosis of Norovirus Infection by Amplification of the RNA-Dependent RNA Polymerase Gene
Source: PLoS One. 2014 Sep 29;9(9):e109876. doi: 10.1371/journal.pone.0109876 (PMC4181653; doi:10.1371/journal.pone.0109876)
Supplement: Table S1 — Sequences and BLAST results of RdRp amplicons in 33 samples false-positive for norovirus. (PDF) [file pone.0109876.s001.pdf]

Sample No.	Sequence	Accession	Description	Ident
1	TAAACTACTTGTGTTCTAGGATAGTTTAGAGGGACTCATTCCCTGCTATCGTGGGTGAGATGTCTATAAAAAGGACAACCAGTGGGCGATGGAATTCCAA	AC021581.10	Homo sapiens chromosome 17, clone RP11-956N15, complete sequence	96%
2	GAATGTATTGTGTCTACGCATAGTTTAGAGGGACTCATTCCCTGCTATCTGGGTGAGATGTCTATGAAAAGGACAACCAGTGGGCGATGGAATTCCAA	AC021581.10	Homo sapiens chromosome 17, clone RP11-956N15, complete sequence	96%
3	GACACCCTTGTGTCTACGTATAGTTTAGAGGGACTCATTCCCTGCTATCTGGGTGAGATGTCTATGAAAAGGACAACCAGTGGGCGATGGAATTCCAA	AC021581.10	Homo sapiens chromosome 17, clone RP11-956N15, complete sequence	96%
4	CAACCGTTTGTGTCTCACGGATAGTTTAGAGGGACTCATTCCCTGCTATCGTGGGTGAGATGTCTATAAAAAGGACAACCAGTGGGCGATGGAATTCCAA	AC021581.10	Homo sapiens chromosome 17, clone RP11-956N15, complete sequence	96%
5	CGGACTATTTGTGCTCTGAGGGATAGTTTAGAGGGACTCATTCCCTGCTATCGTGGGTGAGATGTCTATGAAAAGGACAACCAGTGGGCGATGGAATTCCAAAG	AC007881.4	AC clone RP11-467P9 from 2, co	96%
6	CAATTTCTTGTGTCTAGGATAGTTTAGAGGGACTCATTCCCTGCTATCGTGGGTGAGATGTCTATGAAAAGGACAACCAGTGGGCGATGGAATTCCAA	AC021581.10	Homo sapiens chromosome 17, clone RP11-956N15, complete sequence	97%
8	TACACGCCTGTGTTCTAGGTATAGTTTAGAGGGACTCATTCCCTGCTATCTGGGTGAGATGTCTATGAAAAGGACAACCAGTGGGCGATGGAATTCCAA	AC115991.4	Homo sapiens chromosome 11, clone CTC-342M10, complete sequence	93%
9	CGAACGTTTGTGTTCTAGGCATAGTTTAGAGGGACTCATTCCCTGCTATCGTGGGTGAGATGTCTATGAAAAGGACAACCAGTGGGCGATGGAATTCCAA	AC007881.4	AC clone RP11-467P9 from 2, co	95%

10	AATCTGGCTTTCTGATCTGACGCTAGTTTAGAGGGACTCATTCCCTGCTATCGTGGGTGAGATGTCTATGAAAAGGACAACCAGTGGGCGATGGAATTCCAA	AC021581.10	Homo sapiens chromosome 17, clone RP11-956N15, complete sequence	97%
11	CACACTCTTGTGTCTACGATAGTTTAGAGGGACTCATTCCCTGCTATCTGGGTGAGATGTCTATGAAAAGGACAACCAGTGGGCGATGGAATTCCAA	AC021581.10	Homo sapiens chromosome 17, clone RP11-956N15, complete sequence	96%
12	TACCAGTCTCGTTGTTTTACGATAGTTTAGAGGGACTCATTCCCTGCTATCTGGGTGAGATGTCTATGAAAAGGACAACCAGTGGGCGATGGAATTCCAA	AC021581.10	Homo sapiens chromosome 17, clone RP11-956N15, complete sequence	96%
13	GTAGGGTATTGTCATCTAGGGATAGTTTAGAGGGACTCATTCCCTGCTATCTGGGTGAGATGTCTATGAAAAGGACAACCAGTGGGCGATGGAATTCCAA	AC021581.10	Homo sapiens chromosome 17, clone RP11-956N15, complete sequence	95%
14	CACACTACTTGTGTTCTAGGATAGTTTAGAGGGACTCATTCCCTGCTATCTGGGTGAGATGTCTATGAAAAGGACAACCAGTGGGCGATGGAATTCCA	AC115991.4	mosome 11, clone CTC-342M10,	92%
15	TACCGTCTTGTTCTTTTACGTAATAGTTTAGAGGGACTCATTCCCTGCTATCGTGGGTGAGATGTCTATGAAAAGGACAACCAGTGGGCGATGGAATTCCAA	AC021581.10	Homo sapiens chromosome 17, clone RP11-956N15, complete sequence	97%
17	GACTCCACTTGTGTCTAGGCATAGTTTAGAGGGACTCATTCCCTGCTATCTGGGTGAGATGTCTATGAAAAGGACAACCAGTGGGCGATGGAATTCCAA	AC021581.10	Homo sapiens chromosome 17, clone RP11-956N15, complete sequence	96%
18	GAGTATAGTGTCTACGATAGTTTAGAGGGACTCATTCCCTGCTATCTGGGTGAGATGTCTATGAAAAGGACAACCAGTGGGCGATGGAATTCCAA	AC021581.10	Homo sapiens chromosome 17, clone RP11-956N15, complete sequence	96%
19	GACTCTCTTGTGTCTAGGATAGTTTAGAGGGACTCATTCCCTGCTATCTGGGTGAGATGTCTATGAAAAGGACAACCAGTGGGCGATGGAATTCCAA	AC021581.10	Homo sapiens chromosome 17, clone RP11-956N15, complete sequence	96%

20	CAATCTCTTGTGTCTACGATAGTTTAGAGGGACTCATTCCCTGCTATCTGGGTGAGATGTCTATGAAAAGGACAACCAGTGGGCGATGGAATTCCAA	AC021581.10	Homo sapiens chromosome 17, clone RP11-956N15, complete sequence	96%
21	GTAGCTAGTGTCATCTGAGGCATAGTTTAGAGAGGACTCATTCCCTGCTATCGTGGGTGAGATGTCTATGAAAAGGACAACCAGTGGGCGATGGAATTCCAA	AC007881.4	AC clone RP11-467P9 from 2, co	96%
22	AAAACCTCTTGTGTCTATGATAGTTTAGAGGGACTCATTCCCTGCTATCGTGGGTGAGATGTCTATGAAAAGGACAACCAGTGGGCGATGGAATTCCAA	AC021581.10	Homo sapiens chromosome 17, clone RP11-956N15, complete sequence	97%
23	GACTTTTTGTGTCTGGGCATAGTTTAGAGGGACTCATTCCCTGCTATCTGGGTGAGATGTCTATGAAAAGGACAACCAGTGGGCGATGGAATTCCAA	AC115991.4	mosome 11, clone CTC-342M10,	91%
24	GGGCTATTGTCATCTGGGGATAGTTTAGAGGGACTCATTCCCTGCTATCGTGGGTGAGATGTCTATGAAAAGGACAACCAGTGGGCGATGGAATTCCAA	AC021581.10	Homo sapiens chromosome 17, clone RP11-956N15, complete sequence	96%
25	CACTCGTCTTGTGTCTAGGCATAGTTTAGAGGGACTCATTCCCTGCTATCTGGGTGAGATGTCTATGAAAAGGACAACCAGTGGGCGATGGAATTCCAA	AC021581.10	Homo sapiens chromosome 17, clone RP11-956N15, complete sequence	96%
26	GACCCTCTTGTGTCTAGGCATAGTTTAGAGGGACTCATTCCCTGCTATCTGGGTGAGATGTCTATGAAAAGGACAACCAGTGGGCGATGGAATTCCAA	AC021581.10	Homo sapiens chromosome 17, clone RP11-956N15, complete sequence	96%
27	GGACCGTCAGTGCTCTCAGGGATAGTTTAGAGGGCTCATTCCCTGCTATCTGGGTGAGATGTCTATGAAAAGGACAACCAGTGGGCGATGGAATTCCAAAG	AC115991.4	Homo sapiens chromosome 11, clone CTC-342M10, complete sequence	93%
30	CACCCTCCTCGTTGTTCTAAGAAAGTTTAGAGGGATCATTCCCTGCTATCGTGGGTGAGATGTCTATGAAAAGGACAACCAGTGGGCGATGGAATTCCAA	AC021581.10	Homo sapiens chromosome 17, clone RP11-956N15, complete sequence	92%

31	TACATACCGTTGTTCTACGATAGTTTAGAGGGCTCATTCCCTGCTATCTGGGTGAGATGTCTATGAAAAGGACAACCAGTGGGCGATGGAATTCCAA	AC021581.10	Homo sapiens chromosome 17, clone RP11-956N15, complete sequence	95%
32	ATAGAGTTGCGTCATCTCATGGATAGTTTAGAGGGACTCATTCCCTGCTATCATGGGTGAGATGTCTATGAAAAGGACAACCAGTGGGCGATGGAATTCCAA	AC115991.4	Homo sapiens chromosome 11, clone CTC-342M10, complete sequence	97%
33	TCCCGCCTCGTTGTTCAAGAAAGTTTATAGGGACTCATTCCCTGCTATCTGGGTGAGATGTCTATAAAAAGGACAACCAGTGGGCGATGGAATTCCAA	AC083906.23	P11-93K22 (Roswell Park Cance	92%
34	CACCGCTTTGTGTCTAGGATAGTTTAGAGGGACTCATTCCCTGCTATCTGGGTGAGATGTCTATGAAAAGGACAACCAGTGGGCGATGGAATTCCAA	AC021581.10	Homo sapiens chromosome 17, clone RP11-956N15, complete sequence	96%
35	TACACCACCGTTGTCTAGGATAGTTTAGAGGGCTCATTCCCTGCTATCTGGGTGAGATGTCTATGAAAAGGACAACCAGTGGGCGATGGAATTCCAA	AC021581.10	Homo sapiens chromosome 17, clone RP11-956N15, complete sequence	95%
36	AAACCTCTTGTGTCTAGGCATAGTTTAGAGGGACTCATTCCCTGCTATCGTGGGTGAGATGTCTATGAAAAGGACAACCAGTGGGCGATGGAATTCCAA	AC021581.10	Homo sapiens chromosome 17, clone RP11-956N15, complete sequence	97%
37	TAGGGGTTCGTTATCTGGGCATAGTTTAGAGGGACTCATTCCCTGCTATCGTGGGTGAGATGTCTATGAAAAGGACACCCAGTGGGCGATGGAATTCCAA	AC021581.10	Homo sapiens chromosome 17, clone RP11-956N15, complete sequence	95%
